# Scirrhous Carcinoma of the Bladder

**Published:** 1882-01

**Authors:** Geo. M. Parkinson


### III.—SCIRRHOUS CARCINOMA OF THE BLADDER.

REPORTED BY GEO. M. PARKINSON, D.V.S.

Grey gelding, æt. 16 to 18. Thin in flesh. This animal had been in poor health for one year previous to death. During the last year of life he was subject to repeated attacks of colic. The horse was turned out to grass.

About three years prior to death the gelding had turns of standing with outstretched limbs, apparently straining with a desire to micturate. From these symptoms, however, he apparently recovered in a few weeks and experienced no further trouble until one year before death.

Prior to October 14th, 1881, the horse had been treated by a so-called veterinary surgeon, not a graduate of any medical school.

October 14th I first saw the animal and learned that the only previous treatment had been diuretics, probably some of the bromide salts.

Symptoms: Pulse 40 and very feeble; respiration not disturbed; abdomen distended, the animal having just drank a large quantity of water. There was an œdematous swelling of the sheath and of the tissue on both sides of the linea-alba as far forward as the posterior limit of the sternum. There was a discharge from the nostrils; the visible mucous membranes of a bluish or copper color; appetite poor. I noticed that the animal made frequent attempts to micturate, passing with each effort only a small quantity of urine, which often contained small blood clots These phenomena evidently caused the horse great pain.

I made a rectal examination, but failed to find the distended bladder as normally recognized in rectal examinations, but instead there was a large mass or tumor of the bladder. This neoplasm lay to the right of the median line just anterior to the sympathis pubis. Upon compressing the tumor the horse groaned as if from pain and simultaneously passed a small quantity of urine containing several small coagula. I diagnosticated malignant growth of the bladder, probably carcinotamous in character, and gave a bad prognoses. No special treatment was instituted further than rest in open pasture.

October 25th, the amimal became so weak that, when down, he could not regain his feet, and consequently was killed.

Necropsy, a few hours after death. The *abdominal* cavity was well filled with a fluid having a strong uriniferous odor. Both viceral and parietal layers of the peritoneum were studded with tubercle-like masses, varying in size, and probably representing infiltrated retroperitoneal glands. The mesentery and omentum appeared as solid masses, replaced or formed by these new formations. The liver and spleen were both much enlarged, and also had these small neoplasms upon the surface, resembling the omental growth, but in neither case involving the gland proper. Upon microscopic examination the liver was found to be pygmented and slightly cirrhotic The stomach was full of fluid which contained a little grass; the mucous membrane of a deep yellow color, probably post mortem biliary staining. The inguinal, as well as the retroperitoneal glands were enlarged, and apparently by extension of the new growth.

The genito-urinary tract and the glandular enlargements were sent to the School of Histology in connection with the Columbia Veterinary College and School of Comparative Medicine, for examination by Prof. Porter, who rendered the following report: When the genito-urinary tract was opened, the bladder was found to be nearly closed by a papillary projection springing from the right side. This papillary or cauliflower excresence was ulcerated at points. In the wall of the bladder and to the right there was a large hard mass. The whole pelvic peritoneum was studded with small tumors. A large portion of the omentum was made up of a mass of these small tumors, ranging in size from a pea to that of an English walnut. The microscopical examination revealed the same appearance as we commonly find in the scirrhous carcinoma; that is, irregular alveolar spaces filled in an irregular manner with large nucleated epithelial corpuscles.

